# Application of the Ridden Horse Pain Ethogram to Horses Competing in British Eventing 90, 100 and Novice One-Day Events and Comparison with Performance

**DOI:** 10.3390/ani12050590

**Published:** 2022-02-25

**Authors:** Sue Dyson, Danica Pollard

**Affiliations:** 1 The Cottage, Church Road, Market Weston, Diss, Suffolk IP22 2NX, UK; 2 The Rodhams, Rodham Road, Christchurch, Wisbech, Cambridgeshire PE14 9NU, UK; drdee.pollard@gmail.com

**Keywords:** ridden behaviour, lameness, canter, noseband, bit, spurs

## Abstract

**Simple Summary:**

The Ridden Horse Pain Ethogram (RHpE) comprises 24 behaviours; a RHpE score ≥8 reflects the presence of musculoskeletal pain. An association between the RHpE score and performance has been shown for 5* three-day event horses. The aim of the study was to apply the RHpE to horses performing a dressage test at British Eventing (BE) 90, 100 and Novice one-day events and to compare the scores with competition results. Data were collected for 1010 competition starts. Overall, the most frequent (median) RHpE score was 4/24. The median RHpE score was higher (5/24) for BE 90 competitors, compared with 100 (4/24) and Novice (3.5/24). Horses placed first, second or third had a lower median RHpE score (2/24) compared with other horses which completed. The proportion of horses with a RHpE score ≥8/24 was lowest (2%) in those placed first to third, followed by horses with lower finish placings (9.9%), and highest in those that were eliminated, retired or withdrawn (11.3%). The overall low median RHpE score supports the social licence to compete, but 9% of starters had a RHpE score ≥8/24, which merits concern. Veterinary investigation of these horses and appropriate treatment and management may improve both welfare and performance.

**Abstract:**

The Ridden Horse Pain Ethogram (RHpE) was applied to 1010 competition starts at British Eventing (BE) 90, 100 and Novice one-day events and compared with performance. The overall median RHpE score was 4/24 (IQR 2,6; range 0,12). There were moderate positive correlations between RHpE scores and dressage penalties (Spearman’s rho = 0.508, 0.468, 0.491, all *p* < 0.001 for BE 90, 100 and Novice, respectively). There were weak positive correlations between RHpE scores and final placings (Spearman’s rho = 0.157, *p* = 0.033, BE90; rho = 0.263, *p* < 0.001, BE 100; rho = 0.123, *p* = 0.035, Novice). In showjumping, 1.7% of starters were eliminated or retired, compared with 9.8% of cross-country starters. Horse or rider falls occurred in 2.6% of cross-country starts. Horses placed first, second or third had lower median RHpE scores (2/24, IQR 1,4; range 0,8) than other horses that finished (*p* < 0.001), those that were eliminated or retired (*p* < 0.001) or were withdrawn (*p* < 0.001). The RHpE score was ≥8/24 for 9.3% of starters; horses with a RHpE score ≥8/24 had higher total penalty scores (*p* < 0.001) than horses with a RHpE score <8/24. The overall low median RHpE score supports the social licence to compete, but 9% of starters had a RHpE score ≥8/24. Investigation and treatment of these horses may improve both welfare and performance.

## 1. Introduction

A Ridden Horse Pain Ethogram (RHpE) comprising 24 behaviours (Table S1) was developed [1], and it was shown that a RHpE score of ≥8/24 is likely to reflect the presence of musculoskeletal pain [1,2,3,4,5,6]. In a previous pilot study assessing horses (*n* = 35) warming up for dressage at a 4* (now 5*) three-day event in 2018, horses with a RHpE score of ≥7 were more likely to be eliminated or retire in the cross-country phase than horses with a RHpE score <7 [7]. The pilot study highlighted that in a cohort of highly trained event horses competing at 5* level, it may be useful to use a slightly lower total RHpE score as an indicator of possible influence on performance, rather than the score of ≥8/24, previously identified as a reliable score for differentiating sports horses with and without musculoskeletal pain.

The RHpE was subsequently applied to all horses (*n* = 137) competing at two 5* three-day events in 2019 [8]. There was a significant association between the RHpE score during warm-up for dressage and both dressage penalties and final placing. Horses with a RHpE score of ≥7 were more likely to be eliminated or retire during cross-country than horses scoring <7. There was an association between lameness or gait abnormalities in canter and a RHpE score ≥7. There was good consistency of results for horses which competed at both events. It was concluded that the use of the RHpE may help to identify horses which might benefit from investigation and treatment to both improve performance and enhance equine welfare.

The RHpE has also been applied to video recordings of horses competing in Grand Prix dressage competitions at elite World Cup level [9] and sub-elite level [10]. There was a negative correlation between the RHpE score and the judges’ total percentage scores.

The median RHpE score for non-lame three-day event horses was 3/24 (range 0, 9), whereas horses which showed transient lameness or gait abnormalities in canter had a significantly higher median RHpE score of 5/24 (range 1, 9) [8]. Elite World Cup Grand Prix dressage horses had a median RHpE score of 3/24 (range 0, 7) [9], whereas sub-elite Grand Prix horses had significantly higher scores (Hickstead Rotterdam Challenge median 4/24 (range 0, 8); British Dressage National Championships median 6/24 (range 1, 9), in association with a higher frequency of occurrence of lameness or abnormalities of canter [10].

British Eventing (BE) one-day events comprise dressage, showjumping and cross-country phases, with the maximum height of cross-country fences being 0.90 m, 1.0 m and 1.10 m for BE 90, 100 and Novice classes, respectively [11]. For BE 90 competitions, horses must be at least 132 cm in height and 5 years of age (4 years of age after 1st July); for BE 100 and Novice competitions horses must be at least 142 cm in height and 5 years of age. A horse may be withdrawn from the competition before the start of any phase. A horse fall or unseated rider in any phase results in elimination. In the dressage phase, a rider may be eliminated because of three errors of course, the appearance of blood on the horse or severe lameness. In the showjumping phase, three cumulative refusals or resistance for >20 s result in elimination. In the cross-country phase, three refusals at a single fence or three (four at BE 90 or 100) cumulative refusals result in elimination. In the dressage phase, the judge awards a score of 0 (not executed) to 10 (excellent) for each movement, and four sets of ‘collective marks’ for overall quality of paces, impulsion, submission and rider position and effectiveness, for a maximum total of 200. The summed total expressed as a percentage is subtracted from 100 to give the penalty score. Penalties are awarded for knocking a fence down (4), run outs or refusals (4 and 8 respectively, for first and second refusals in showjumping; 20 and 40 respectively, for first and second refusals in cross-country), or for exceeding (showjumping and cross-country) or going under (cross-country) the optimum time. The optimum cross-country speeds are 450 m/min, 475 m/min and 520 m/min for BE 90, 100 and Novice, respectively. A rider may elect to retire a horse during any phase, usually because it is not performing well.

The objectives of the study were: 1. to apply the RHpE to horses competing in the dressage phase at BE 90, 100 and Novice one day events; 2. to document the frequency of occurrence of each behaviour of the RHpE and 3. to compare the RHpE scores with performance. It was hypothesised that there would be an association between higher RHpE scores and poorer performance results.

## 2. Materials and Methods

### 2.1. Data Acquisition

The study was approved by the Royal College of Veterinary Surgeons Ethics Review Panel (2020–26); data were collected at public events, therefore informed rider consent was not required. Data were collected from a convenience sample of BE one-day events at BE 90, 100 and Novice levels. At each level the horses performed a set BE dressage test in a 40 m × 20 m arena. This included halt, medium walk, free walk on a long rein, working trot and working canter at all levels, and medium trot and canter, counter canter ± rein back and leg yield at Novice level. The tests were selected by the event organiser from those available at each BE level; the duration of each test was approximately five minutes. Each section was judged by a single trained judge, listed by British Dressage as qualified to officiate at that level. All horses in each randomly selected section were assessed. Horses were identified by the rider’s number. At preselected events, with the cooperation of the organisers, competitors were offered the opportunity to opt out of the study prior to the day of the competition, but none elected to do so. Ridden Horse Pain Ethogram data were collected by a single trained assessor (SD, an equine veterinarian with 42 years of experience of lameness investigation) who stood approximately 1–3 m from the dressage arena at the MC corner (i.e., 10 m to the left of the official judge who was positioned on the centre line). The RHpE data were recorded manually (binary yes/no scoring) on individual purpose-designed score sheets.

Additional subjective data were recorded (for example, forelimb lameness, short stepping forelimb gait, hindlimb lameness, lack of hindlimb impulsion and engagement in trot and/or canter, canter lacks a suspension phase). Behaviour 20 of the RHpE includes both repeated bilateral hindlimb toe drag or repeated stumbling. The presence of bilateral hindlimb toe drag or single or repeated stumbling were also recorded independently. For those Novice tests that included rein back, behaviours such as head behind vertical, mouth opening with separation of the teeth, head tossing, ears back and refusal to step backwards were also recorded independently. These behaviours were not included in the RHpE scores if not fulfilling the RHpE definitions (for example, head above vertical ≥30°, but <10 s).

Snaffle bridles were required for dressage; the use of spurs was optional. The type of noseband used (cavesson, crank cavesson, flash, crank flash, Micklem, grackle, drop), the use of a nose net and whether the rider used spurs were documented. Weather conditions, the footing and the levelness of the arena were also recorded.

Dressage, showjumping, cross-country and total penalties, and final place were collected from each event’s website. Reasons for elimination were recorded. Each horse’s age, breed and sex information were collected from the BE website.

### 2.2. Data Analysis

Data were recorded in an electronic spreadsheet (Microsoft Excel, version 2010; Microsoft Corporation, Redmond, WA, USA) and statistical analysis was carried out using commercial statistical software (STATA: IC version 13; StataCorp. LLC. 2017. Stata Statistical Software: Release 15. College Station, TX, USA).

The distribution of continuous variables (horse age, dressage, showjumping, cross-country and overall penalties) was formally evaluated using the Shapiro-Wilk test, in combination with visual assessment of histograms, with overlaid kernel density plots. All continuous variables were determined not to have a normal distribution (Shapiro-Wilk *p*-value < 0.05) and alongside ordinal variables (RHpE score and final placing) were described as medians with interquartile range (IQR) and range (minimum to maximum). Categorical variables (horse sex and breed, noseband type, use of spurs, presence of gait abnormalities and RHpE behaviours (yes/no), completion status (placed in top three, unplaced, eliminated/retired or withdrawn), elimination/retirement during a specific phase of the competition and competition level (BE 90, 100 or Novice) were summarised as proportions and expressed as percentages. Associations between horse signalment and competition level were described but not statistically assessed. This is because some horses had repeated observations within and across levels, and the focus was on assessing behaviour at competition starts rather than at horse level.

Relationships between categorical variables were assessed using the Chi-square (*χ*^2^) or Fisher’s exact test when observed counts in any comparison group were <5. These included the presence of gait abnormalities and RHpE behaviours, completion status, elimination/retirement during a specific phase of the competition and competition level. Where significant relationships were identified between categorical variables in 2 × 3 contingency tables, an additional Cramer’s V test was calculated to assess the strength of the association/estimate effect size [12]. Qualitative interpretation of Cramer’s V was performed according to Rea and Parker [13], with <0.20 signifying a weak association, ≥0.20 to <0.40 signifying a moderate association and ≥0.40 signifying a strong association. The Bonferroni correction was used to adjust for multiple comparisons where the significance level of the α test (*p* = 0.05) was divided by the number of tests/comparisons.

The Mann-Whitney *U* test was used to assess the relationship between continuous/ordinal variables and categorical variables (e.g., overall penalties and RHpE category [<8 vs. ≥8]). Overall median differences in RHpE scores and competition level and completion status were assessed using the Kruskal-Wallis test, with a further post-hoc Dunn’s test with Sidák adjustment to assess pairwise comparisons between groups.

Correlations between RHpE scores and dressage, showjumping and cross-country penalties and overall placing at each competition level were assessed using the Spearman rank correlation coefficient.

## 3. Results

### 3.1. Overall Results

Data were collected at 20 competition days between 17 April 2021 and 29 October 2021 at venues in Bedfordshire, Cambridgeshire, Leicestershire, Lincolnshire, Norfolk, Northamptonshire, Suffolk, Surrey and Sussex. This included 34 sections: Novice *n* = 12 (356 competition starts), BE 100 *n* = 15 (450 competition starts) and BE 90 *n* = 7 (204 competition starts), comprising a total of 1010 competition starts by 841 horses and 708 riders. The dressage tests took place on grass arenas for 20 sections, including 588 (58.2%) tests, or on all-weather arenas at two venues for 14 sections, including 442 (41.8%) tests. The weather was variable among venues and times of day, and included wind, rain, sun, cool and occasionally warm/hot.

#### 3.1.1. Horse Data

The median age for all horses (*n* = 841) was nine years (IQR 7, 11; range 5, 22), with 563 (66.9%) geldings, 5 (0.6%) stallions and 273 (32.5%) mares. Breeds included Irish Sports Horse *n* = 335 (39.8%), Warmblood *n* = 188 (22.4%), Warmblood cross *n* = 153 (18.2%), Thoroughbred or Thoroughbred cross *n* = 9 (1.1%), Other crossbred/unknown *n* = 103 (12.3%), and Pony *n* = 53 (6.3%).

#### 3.1.2. Nosebands and Spurs

Data for noseband type and the use of spurs were not available for the first section evaluated, *n* = 28. Noseband types (*n* = 982 of competition starts) included: grackle *n* = 248 (25.3%), crank flash *n* = 231 (23.5%), flash *n* = 164 (16.7%), Micklem *n* = 137 (14.0%), crank cavesson *n* = 92 (9.4%), cavesson *n* = 91 (9.3%) and drop *n* = 19 (1.9%). A nose net was used in 7 of 1010 (0.7%) competition starts. Spurs were used in 719/982 (73.2%) of competition starts, but not in 263 (26.8%). Tail swishing in synchrony with the application of spur cues was not observed.

#### 3.1.3. Gait Abnormalities

The frequencies of occurrence of gait abnormalities in trot and canter are summarised in Table 1. Overall, there was a low frequency of occurrence of overt lameness (8.6%), but poor hindlimb impulsion and engagement were observed in 38.1% of competition starts, and canter was abnormal in the majority (61.0%).

Teeth grinding or chomping repeatedly were observed in 62 (6.1%) of competition starts. Rein back was performed at seven competitions (Novice 111 (2010) test) and was performed poorly (relative to the guidelines for dressage judges [11]) in 45/197 (23%) competition starts (not including horses that stepped back crookedly or took an incorrect number of steps). Errors included refusing to step backwards, the front of the head being considerably in front of or behind a vertical position, opening the mouth widely, ears back, tail swishing, head tilt and head tossing. However, the duration of these abnormal behaviours was generally less than the RHpE definition.

#### 3.1.4. Ridden Horse Pain Ethogram Score and Performance

Overall, the median RHpE score was 4 (IQR 2, 6; range 0, 12). The median dressage penalty score (*n* = 1009, because of 1 elimination) was 33 (IQR 30.5, 35.8; range 18.5, 56.8). The median showjumping penalty score (*n* = 980 because of withdrawals, eliminations and retirements) was 4 (IQR 0, 4; range 0, 52). The median cross-country penalty score (*n* = 851 because of withdrawals, eliminations and retirements) was 4 (IQR 0, 14; range 0, 139.2). The median total penalty score was 41.7 (IQR 35.3, 54.8; range 20.5, 170.5).

The median RHpE score for horses placed first, second and third in a section was 2 (IQR 1, 4; range 0, 8), compared with a median RHpE score of 4 (IQR 2, 6; range 0, 12) for all other horses that completed. Horses placed in the top three had significantly lower (*p* < 0.001) median RHpE scores compared with horses which completed but were not placed in the top three.

The median RHpE score for horses eliminated or retired in the showjumping or cross-country phases (*n* = 112) was 4.5 (IQR 2, 6; range 0, 12). One horse was eliminated in the dressage phase. The median RHpE score for horses withdrawn (*n* = 47) was 6 (IQR 3, 7; range 0, 8). Twelve horses were withdrawn before show jumping and 35 were withdrawn before cross-country. Seventeen of 997 (1.7%) showjumping starters were eliminated or retired. Ninety-four of 945 (9.8%) cross-country starters were eliminated or retired. There was a total of 25 unseated riders (2.5%), including two in the showjumping phase. There were 25 horse or rider falls in the cross-country phase, representing 2.6% of cross-country starters. This included two horse falls (0.2% of cross-country starters).

There were significant differences in the median RHpE scores between horses placed in the top three and unplaced horses (*p* < 0.001), horses that were eliminated/retired (*p* < 0.001) and horses that were withdrawn (*p* < 0.001). There was also a significant difference in the median RHpE scores between unplaced horses and withdrawn horses (*p* = 0.04). However, no differences in median RHpE scores were identified between unplaced horses and eliminated/retired horses (*p* = 0.687) nor between eliminated/retired and withdrawn horses (*p* = 0.297).

The RHpE score was <8 for 916 (90.7%) competition starts but was ≥8 for 94 (9.3%) competition starts. The proportion of horses with a RHpE score ≥8 was lowest in those placed first to third (2.0%), followed by horses with lower finish placings (9.9%), and highest in those that were eliminated, retired or withdrawn (11.3%) (*p* = 0.01). Considering horses that completed, horses with a RHpE score ≥8 had significantly higher total penalty scores (median 47.8, IQR 40.2, 62.0; range 31.8, 116.8) than horses with a RHpE score <8 (median 41.1, IQR 34.8, 53.5; range 20.5, 170.5) (*p* < 0.001).

### 3.2. Results Presented for Each Level Independently

#### 3.2.1. Horse Data

There was a high proportion of Warmblood horses in BE 100 and Novice sections relative with BE 90, while the BE 90 sections had a relatively high proportion of ponies (Table 2).

Age and sex data are summarised in Table 3. The median age was highest for horses competing at BE 90 level. Geldings predominated at all levels.

#### 3.2.2. Gait Abnormalities

The distribution of gait abnormalities at each level is summarised in Table 1. There was a significant but weak relationship (*χ*^2^ *p* < 0.001; Cramer’s V < 0.20) between competition level and forelimb lameness, hindlimb lameness and lack of hindlimb impulsion and engagement, and a moderate association between competition level and abnormal canter (Cramer’s V 0.23). The frequency of occurrence of these gait abnormalities was highest at BE 90 and lowest at BE Novice competition level.

#### 3.2.3. Ridden Horse Pain Ethogram

**Figure 1:**
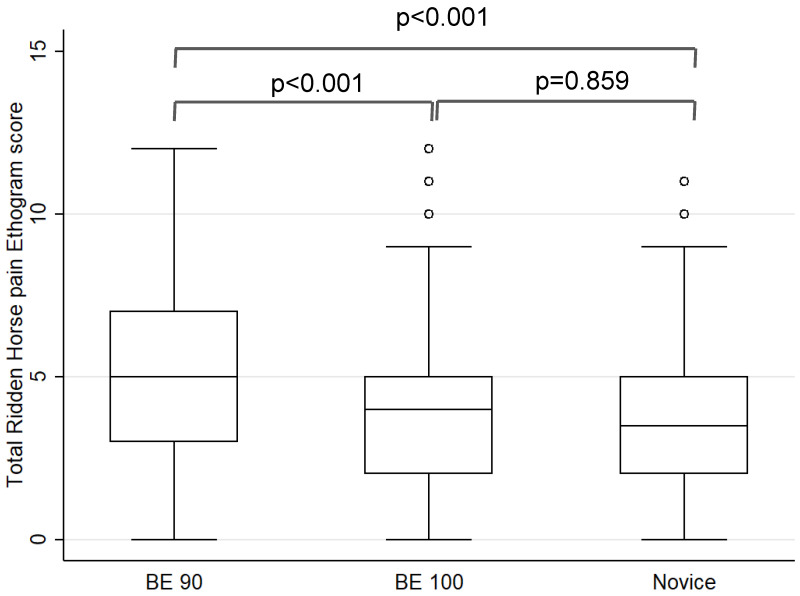
Box and whisker plots for the total Ridden Horse Pain Ethogram scores during the dressage phase compared with competition level (British Eventing [BE] 90, 100 and Novice) for 1009 competition starts (one horse at Novice level was eliminated in the dressage). Boxes represent medians and interquartile ranges; whiskers represent the range and individual points outliers. The median RHpE scores were significantly higher for BE 90 compared with both BE 100 and Novice.

When considering each level of competition separately, at BE 90 the median RHpE score was 5 (IQR 3, 7; range 0, 12), compared with a median RHpE score of 4 (IQR 2, 5; range 0, 12) at BE 100 and a median RHpE score of 3.5 (IQR 2, 5; range 0, 11) at Novice. There were significant differences in the median RHpE scores between BE 90 and both BE100 (*p* < 0.001) and Novice (*p* < 0.001), but not between BE 100 and Novice (*p* = 0.859) (Figure 1).

The frequency of occurrence of the 24 behaviours of the RHpE overall and at each competition level are documented in Table 4. There was a significant and weak to moderate relationship (*χ*^2^ *p* ≤ 0.001; Cramer’s V < 0.30) between 10 of the RHpE behaviours and competition level, with repeated movement of the head up and down, head in front of the vertical, repeated side to side movement of head, ears behind vertical, an intense stare, mouth opening with separation of the teeth, bit pulled through to one side, repeatedly crooked and repeated bilateral hindlimb toe drag and/or stumbling being most frequent in BE 90 competitions. Spontaneous change of gait was most frequently observed at Novice competitions.

There was a significant and moderate relationship (*χ*^2^ *p* < 0.001; Cramer’s V 0.27) between stumbling or bilateral hindlimb toe drag and competition level, with frequency highest at BE 90 competitions (50.5%) followed by BE 100 competitions (45.1%), and frequency lowest at Novice competitions (20.2%). Bilateral hindlimb toe drag or repeated stumbling was observed more frequently on all weather surfaces (43.6%) compared with grass (37.4%) (*χ*^2^ *p* = 0.001).

#### 3.2.4. Competition Performance

The proportions of completions, eliminations in any phase, retirements (in showjumping or cross-country) and withdrawals (before show jumping or cross-country) are summarised in Table 5. A relationship between completion status and competition level was not identified (*χ*^2^ *p* = 0.06).

The proportions of eliminations or retirements in the showjumping and cross-country phases at each competition level are summarised in Table 6. A relationship between discipline and eliminations or retirements for each competition level was not identified (showjumping *χ*^2^ *p* = 0.386; cross country *χ*^2^ *p* = 0.502).

#### 3.2.5. British Eventing 90 Level, *n* = 204

**Figure 2:**
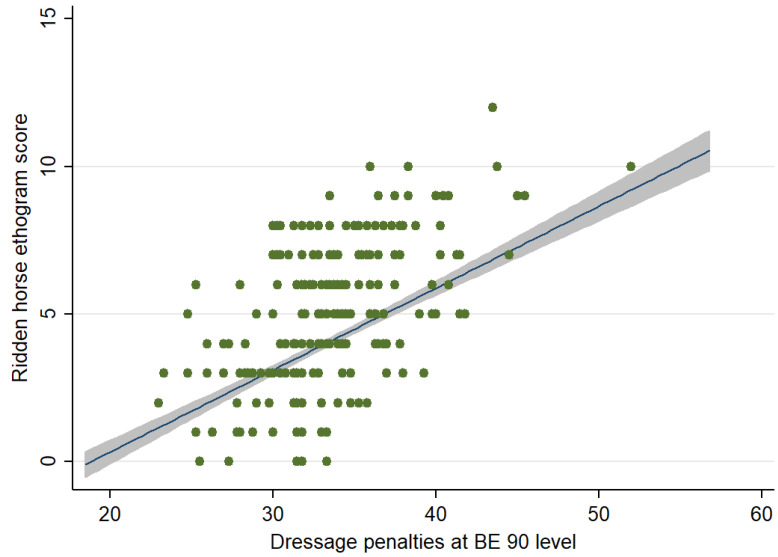
Relationship between the Ridden Horse Pain Ethogram (RHpE) scores and dressage penalty scores for competition starts at British Eventing 90 level (*n* = 204). The green dots represent individual data points, the blue line is the best linear predicted fit and the grey area represents the corresponding 95% confidence interval. There was a moderate positive correlation (Spearman’s rho = 0.508, *p* < 0.001) between the RHpE scores and the dressage penalty scores.

When considering the relationship between RHpE scores and performance, there was a moderate positive correlation (Spearman’s rho = 0.5083, *p* < 0.001) between the RHpE scores and the dressage penalty scores (Figure 2).

**Figure 3:**
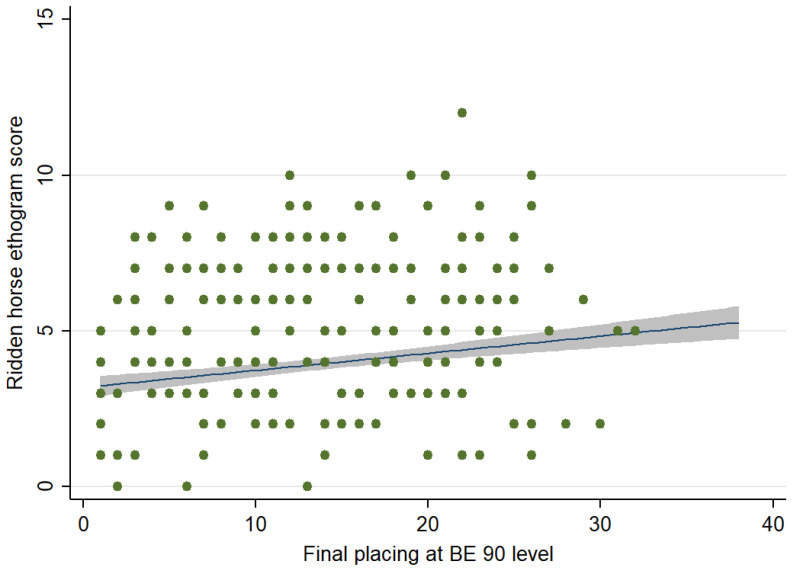
Relationship between the Ridden Horse Pain Ethogram (RHpE) scores and final placings at British Eventing 90 level. The green dots represent individual data points, the blue line is the best linear predicted fit and the grey area represents the corresponding 95% confidence interval. There was a weak positive correlation (rho= 0.157, *p* = 0.033) between the RHpE scores and final placings for 182 completions.

There was no correlation between RHpE scores and showjumping (Spearman’s rho = 0.069, *p* = 0.327) or cross-country (Spearman’s rho = −0.043, *p* = 0.561) penalties for horses that completed each phase. However, there was a weak positive correlation (Spearman’s rho= 0.157, *p* = 0.034) between the RHpE scores and final placing for 182 completions (Figure 3).

#### 3.2.6. British Eventing 100 Level, *n* = 450

**Figure 4:**
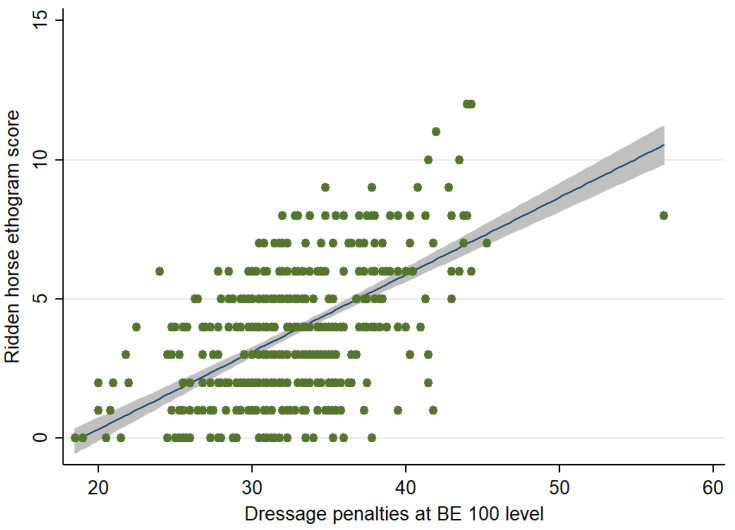
Comparison between the Ridden Horse Pain Ethogram (RHpE) scores and dressage penalty scores for competition starts at British Eventing 100 level (*n* = 450). The green dots represent individual data points, the blue line is the best linear predicted fit and the grey area represents the corresponding 95% confidence interval. There was a moderate positive correlation (Spearman’s rho = 0.468, *p* < 0.001) between the RHpE scores and the dressage penalty scores.

When considering the relationship between the RHpE scores and performance, there was a moderate positive correlation (Spearman’s rho = 0.468, *p* < 0.001) between the RHpE scores and the dressage penalty scores (Figure 4).

**Figure 5:**
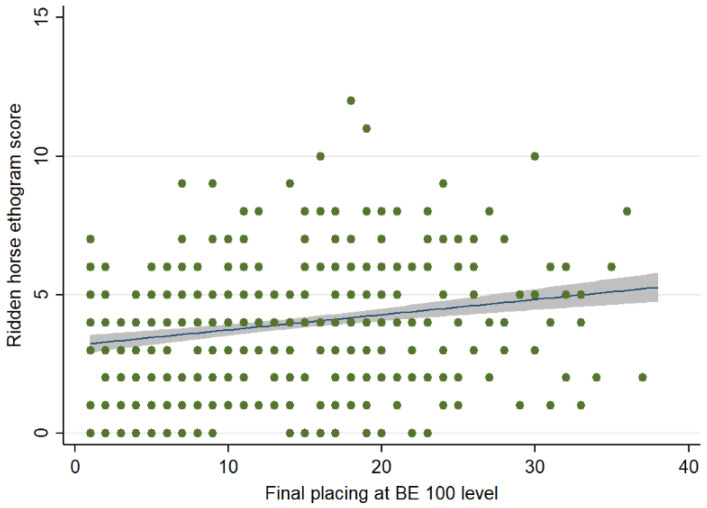
Comparison between the Ridden Horse Pain Ethogram (RHpE) scores and final placings at British Eventing 100 level. The green dots represent individual data points, the blue line is the best linear predicted fit and the grey area represents the corresponding 95% confidence interval. There was a weak positive correlation (rho = 0.263, *p* < 0.001) between the RHpE scores and final placings for 375 completions.

There was no association between the RHpE scores and showjumping (Spearman’s rho = 0.088, *p* = 0.065) or cross-country (Spearman’s rho = 0.098, *p* = 0.057) penalties for horses that completed each phase. However, there was a weak positive correlation (Spearman’s rho = 0.263, *p* < 0.001) between the RHpE scores and final placings for 375 completions (Figure 5).

#### 3.2.7. British Eventing Novice Level, *n* = 356

**Figure 6:**
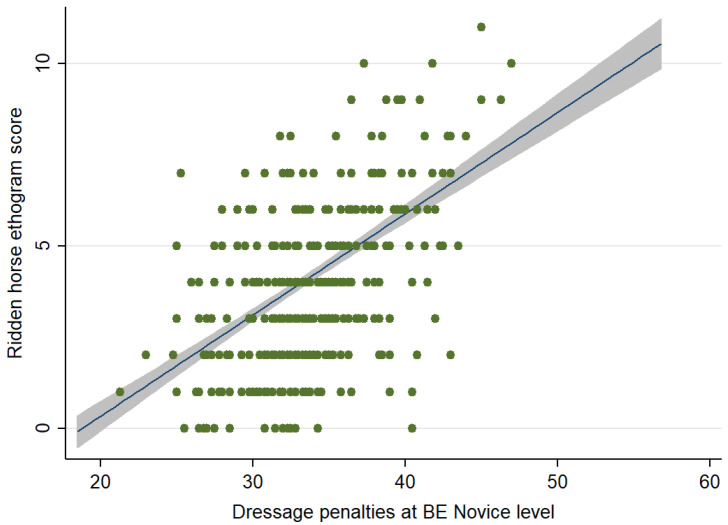
Comparison between the Ridden Horse Pain Ethogram (RHpE) scores and dressage penalty scores for competition starts at British Eventing Novice level (*n* = 355; 1 horse was eliminated). The green dots represent individual data points, the blue line is the best linear predicted fit and the grey area represents the corresponding 95% confidence interval. There was a moderate positive correlation (Spearman’s rho = 0.491, *p* < 0.001) between the RHpE scores and the dressage penalty scores.

When considering the relationship between the RHpE scores and performance at Novice level, there was a moderate positive correlation (Spearman’s rho = 0.491, *p* < 0.001) between the RHpE scores and dressage penalties for 355 competition starts (one horse was eliminated) (Figure 6).

**Figure 7:**
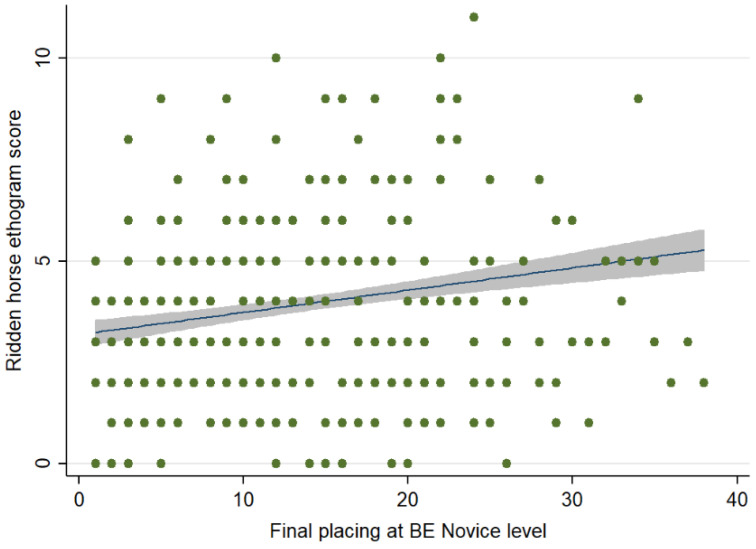
Comparison between the Ridden Horse Pain Ethogram (RHpE) scores and final placings at British Eventing Novice level. The green dots represent individual data points, the blue line is the best linear predicted fit and the grey area represents the corresponding 95% confidence interval. There was a weak positive correlation (Spearman’s rho = 0.123, *p* = 0.035) between the RHpE scores and final placings for 294 completions.

There was no association between the RHpE scores and the showjumping (Spearman’s rho = 0.053, *p* = 0.331) or cross-country (Spearman’s rho = 0.014, *p* = 0.809) penalties for those horses that completed each phase. However, there was a weak positive correlation (Spearman’s rho = 0.123, *p* = 0.035) between the RHpE scores and final placings for 294 completions (Figure 7).

## 4. Discussion

In accordance with our hypothesis, there was a relationship between RHpE scores and performance, with significant correlations between RHpE scores and both dressage penalties and final placings of the horses that completed. Horses that were placed first to third had lower median RHpE scores than other finishers, and horses with RHpE scores ≥8 were over-represented in non-completing and lower-placed horses. However, there was no correlation between the dressage phase RHpE scores and either showjumping or cross-country penalties for those horses which completed. At BE 90 and 100 levels the height of the fences is small, and the course designs are straightforward, with riders often riding more positively when showjumping and riding cross-country than in the dressage phase [14]. Moreover, the release of endorphins and adrenaline when jumping [15,16] may enhance horses’ performances by masking musculoskeletal discomfort. The completion proportion was similar at all competition levels, despite amateur riders predominating at BE 90 level, whereas at BE 100 and Novice levels there was a combination of both amateur and professional riders, including Olympic, European and World Championship level riders. Performance may be influenced by numerous factors including the course, the talent and physical aptitude of the horse and rider [17], as well as musculoskeletal pain.

### 4.1. Frequency of Gait Abnormalities and Level

The frequency of occurrence of lameness (overall 17%, BE 90 32%, 5* three-day events 13%), lack of hindlimb impulsion and engagement (overall 38%, BE 90 48%, 5* three-day events 7%) and abnormalities of canter (overall 61%, BE 90 75%, 5* three-day events 28%) was highest for horses competing at BE 90 level in the current study and higher than previously recorded for horses competing at 5* three-day events [8]. The horses competing at BE 90 also had higher median RHpE scores, probably reflecting discomfort.

The overall high frequency of occurrence of head behind the vertical ≥10° for ≥10 s (59%), poor hindlimb impulsion and engagement (38%) and abnormal canter (61%), often characterised by lack of a suspension phase, also introduces the question of the relative roles of training and riding ability versus discomfort, and of the potential adverse consequences of inappropriate training on musculoskeletal health [18]. Improved gait quality was often seen in medium trot and canter compared with working gaits in Novice tests [14], suggesting that with more positive or less defensive or restrictive riding there was the potential for improvement in gait quality. There was a much larger spectrum of riding ability seen at BE 90 compared with Novice levels, reflecting the higher proportion of professional riders at Novice level [14].

### 4.2. Manifestations of the RHpE and Competition Level

#### 4.2.1. Comparison within Levels

There was a higher frequency of occurrence of some behaviours of the RHpE seen in horses competing at BE 90 level compared with BE 100 and Novice. These included repeated movement of the head up and down or from side to side, the head in front of vertical ≥30° for ≥10 s, ears back ≥5 s, an intense stare ≥5 s, repeated bilateral hindlimb toe drag or repeated stumbling and the bit pulled through to one side repeatedly. This may reflect the higher frequency of gait abnormalities at BE 90 level, which was associated with a higher median RHpE score. It may also, in part, reflect rider skill, as observed in a previous study which compared RHpE behaviours when horses were ridden by two riders of varying skill [19]. There was no difference in the total RHpE scores when ridden by the two riders, but the behaviours exhibited varied according to rider skill. A more skilled rider has a stable phase synchrony with the horse [20,21,22], a more consistent trunk and limb position [23,24], superior ability to control the position of the horse’s head [25] and the ability to create more propulsion [26] compared with less-skilled riders. Less-skilled riders may have less independent control of the arms and hands compared with more skilled riders [21,27], and a lack of ability to steer or straighten the horses with other aids. These factors may contribute to an unstable head position, the bit being pulled through to one side and hindlimb toe drag.

Spontaneous changes of gait were observed more frequently at Novice level than at lower levels. Some of the movements were biomechanically more challenging at Novice level compared with BE 90 and 100, for example counter canter, which may have predisposed to more errors. Spontaneous changes of gait were observed in a similar proportion (17.5%) of sub-elite Grand Prix dressage horses [10] compared with only 8.8% of elite Grand Prix dressage horses [9]. The sub-elite group had higher RHpE scores and a higher proportion of gait abnormalities than the elite Grand Prix horses.

#### 4.2.2. Comparison with 5* Three-Day Events and Grand Prix Dressage

When the overall frequency of occurrence of specific behaviours of the RHpE observed in the current study was compared with horses competing at 5* three-day events [8], clear differences were observed. Head in front of a vertical position ≥30° for ≥10 s, head up and down repeatedly, ears back ≥5 s, bit pulled through repeatedly, moving on three tracks and repeated spontaneous changes of gait occurred more frequently in the lower-level horses. The explanation may be multifactorial, reflecting the higher frequency of pain-related gait abnormalities at the lower levels, an overall lower skill level of riding and inferior training. However, repeated tail swishing was seen more often in the 5* level event horses [8]) compared with horses in the current study, and was also a frequent observation in Grand Prix dressage horses [9,10]. This may be a reaction to stronger application of leg and spur cues by the riders, or the horses experiencing more difficulty with movements requiring a greater level of collection.

Persistent positioning of the head >10° for ≥10 s behind a vertical position was seen with similar frequency in this study (59%) and in 5* three-day event horses (64%) [9], and was also observed in elite (67%) [9] and sub-elite (77%) [10] Grand Prix dressage horses. This is contrary to judging guidelines [28,29] but appears to be inadequately penalised. There are limited scientific data concerning head and neck position and the kinetic and kinematic effects on the limbs and thoracolumbosacral region. The immediate effects of short-term alterations in head and neck position in a small number of non-ridden [30,31] and ridden [32,33,34] riding horses [30] or well-trained dressage horses [31,32,33,34] on a treadmill at walk and trot have been investigated. The data generated cannot be used to determine the long-term effects of regular overground ridden exercise with the head behind vertical at all paces from a young age. However, clinical observations indicate adverse consequences on the optimal development of the pelvic and hindlimb muscles, the abdominal ‘core’ muscles, the muscles of the thoracic sling, the cervical muscle and the epaxial and hypaxial muscles of the thoracolumbosacral regions, and for the establishment of correct movement patterns of the forelimbs, the hindlimbs and the thoracolumbosacral region [18,35,36,37], factors which may have the potential to predispose to injury.

Mouth opening with separation of the teeth for ≥10 s was observed in only 28% of competition starters in the current study, compared with 44% of non-lame sports horses [6], 45% of 5* three-day event horses warming up for dressage [8] and 81% [10] and 68% [9], respectively, of sub-elite and elite dressage horses during Grand Prix tests. Mouth opening may be a non-specific response to musculoskeletal discomfort [1,2] or reflect oral discomfort secondary to the buccal mucosa being pressed against the sharp edges of the teeth [38] or other oral lesions [39], excessive rein tension [40,41], movements of the rider’s hands [42] or the type and size of the bit relative to the size and shape of the horse’s oral cavity and tongue [43,44]. The lower frequency of occurrence of mouth opening in the current study compared with previous studies [6,8,9,10] may reflect the use of only snaffle bits rather than double bridles.

To what extent mouth opening is promoted by, or restricted by, potentially restrictive nosebands or nosebands that are tightened excessively is subject to debate [45,46,47,48,49,50,51], with limited fact-based information concerning pressure effects of nosebands [52,53,54]. In the current study, despite the relatively low frequency of occurrence of mouth opening, the majority of horses (92%) had potentially restrictive nosebands. However, the tightness of the nosebands was not evaluated objectively, nor was there any legislative assessment or control of noseband tightness. In an observational study of 750 competition horses in Ireland, Belgium and the United Kingdom, objectively evaluated noseband tightness was highest in event horses compared with dressage horses and show hunters [47]. It nonetheless seems unlikely that the use of a potentially restrictive noseband was a major causal factor of mouth opening in the current study.

### 4.3. Use of Spurs

Spurs were used in a high proportion (73%) of competition starts in the current study. There is limited documented information about spur use in event horses. In an observational study of 3143 dressage, showjumping, event and endurance horses in competition in Denmark spurs were used in 77%, however event horses and ponies comprised only 3.3% of the study population and it was not possible to determine spur use specifically related to event horses [55]. In an online questionnaire-based study in the United Kingdom with 628 responses, 12 of 33 (36%) event riders used spurs [56]. In a similar Australian-based questionnaire study in 2012 with 1101 respondents, including 50 event riders, overall, 41% of riders reported the regular use of spurs [57].

Contrary to observations in elite Grand Prix dressage horses [9], no tail swishing in synchrony with spur use was observed in the current study. This may reflect either the absence of the application of spur cues or less forceful use of spur cues among the lower-level event riders compared with elite dressage riders.

### 4.4. Failure to Complete

In the current study, the non-completion proportion rose from 11% at BE 90 to 17% at Novice level. This compares with a non-completion proportion of 19% from 42,810 entries across all levels in 2007 [58]. In the current study, horse falls comprised 0.2% of all cross-country starts compared with 0.04% of 576 cross-country starts in a convenience sample of events ranging from Novice one day events to 4* (now 5*) three-day events in 2001 and 2002 [59]. In the latter study, the risk of a horse fall was significantly higher at three-day events compared with one-day events, particularly at Advanced level. In the current study, horse falls or unseated riders comprised 2.6% of all cross-country starters compared with only 0.83% of all starters in BE competitions for the years 1996–1999 [60] and 0.76% in 2000 [61]. In the latter study it was noted that amateur event riders were approximately 20 times more likely to fall than professional riders. The current results appear to reflect a disturbing trend of an increased proportion of unseated riders at the lower levels. These results are consistent with the most recent BE Safety report (2019) [62], which recorded horse falls or unseated riders in 2.4% of approximately 66,000 cross-country starts across all levels (BE 80 to Advanced, including 4* [now 5*] three-day events), with the largest proportion of starts being at BE 90, 100 and Novice levels. In a study of Fédération Equestre Internationale international competitions, including European and World Championships and Olympic Games, from 2008 to 2018, of 187,602 cross-country starts there were 1.5% horses falls and 3.5% unseated riders [63]. There were mildly increased odds of a horse fall (1.1) or unseated rider (1.1) if the dressage penalty score was >50 compared with ≤50.

### 4.5. Social Licence to Compete

The social licence to use horses in competition is increasingly being questioned [64,65,66,67,68]. The overall low median RHpE score observed in the current study supports the continuing use of horses in affiliated eventing. However, a RHpE score ≥8/24 was documented in 9% of competition starts, and this merits attention. Several dressage judges commented in conversation after the event that they considered that some horses looked clearly uncomfortable, but they felt powerless to intervene. Even when overt lameness was observed, judges commented that they felt reluctant to advise competitors to withdraw, although it was within their remit to do so [11], because of previous adverse experiences. On some occasions competitors had sought the advice of the event veterinarian, who only evaluated their horse moving in hand, and no lameness had been observed. This had resulted in complaints to the event organisers about the dressage judges. It must be borne in mind that there is a considerably higher frequency of occurrence of lameness in ridden horses compared with horses assessed in hand [6,69].

Education of riders and coaches/trainers is required to recognise both gait abnormalities that reflect discomfort and ridden horse behaviours that are a manifestation of pain, and to understand the potential consequences of incorrect training on long-term musculoskeletal health. The relationship between RHpE scores and performance highlights the importance of recognition and management of pain for optimising performance. Riders and their coaches/trainers also need to learn to consider all reasons why a horse performed poorly, rather than attribute blame to rider errors, ground conditions, the uncooperative nature of the horse or the difficulty of the course.

### 4.6. Dressage Penalties and RHpE Scores

The dressage tests at BE 90, 100 and Novice level are straightforward, are not biomechanically demanding and should be relatively easy for a pain-free equine athlete that has been trained and ridden correctly. Nonetheless, there was a large range of dressage penalties and RHpE scores, although considerable clustering of dressage penalties, with a large proportion being between 30 and 40 (Figure 2, Figure 4 and Figure 6). This is likely to reflect the limited range of marks used for each movement; a penalty score of 30 equates to a mean mark of 7 (fairly good) (on a scale of 0 [not executed] to 10 [excellent]) per movement, whereas a penalty score of 40 equates to a mean mark of 6 (satisfactory) per movement [28]. Although there was a moderate correlation between the RHpE score and dressage penalties at all competition levels, there were some notable outliers. For example, at BE 100 there was a horse with a RHpE score of 6 (Figure 2) and a dressage penalty score of 27.8, despite lack of hindlimb impulsion and engagement; marked repeated bilateral hindlimb toe drag; mouth opening with separation of teeth ≥10 s; repeated tail swishing and a stiff stilted canter, lacking suspension. This draws into question the accuracy of some judging, as has been previously observed [70,71,72]. According to the guidance to judges, the marks are assessed based on ‘the gaits (‘The trot is free, supple, regular and active. The canter is united, light and balanced’), impulsion (‘…the engagement of the hindquarters, originating from a lively impulsion. The hindquarters are never inactive or sluggish’), and submission (‘…Harmony with rider, lightness of movements and acceptance of the bit with submissiveness/thoroughness without any tension or resistance.’)’ [28,29]. Riders have commented that ‘harsh, forceful training practices were sometimes rewarded by judges’ [16]. It has previously been observed that ‘…despite the rigorous training that judges receive, they do not protect horses from poor riding or poor welfare. This could be addressed by providing better training to allow judges to recognise and mark down behavioural signs that are indicative of conflict or underlying pain’ [67].

### 4.7. Rein Back

Rein back was only included in the dressage test at Novice level, but was executed poorly by 23% of competition starters relative to judging guidelines [28]. Major errors in rein back were also observed in Grand Prix dressage competitions [9,10]. This presumably reflects either inadequate training and practice or conflict behaviour [73]. In a small study (*n* = 32) of dressage horses warming-up before a test, rein back was rarely performed [74]; whether rein back was included in the subsequent test was not documented. However, it is acknowledged that rein back is ‘the severest test of the coordination between driving and restraining influences’ [75] and ‘proof of the degree of suppleness, the action of the rein through the body and obedience’ [76]. The rules indicate clearly that the front of the head should remain vertical and resistance to or evasion of the contact are serious faults [26]. While the movement has clear practical utility, for example being required to open a gate while out hacking, training of this movement in a dressage arena needs to be improved and may be facilitated by early ground work [77,78] and when ridden, accepting one or two steps initially, before progressively asking for more steps [76,79,80,81]. With improved performance of rein back, competitors could gain valuable additional marks.

### 4.8. Limitations of the Study

The study had some limitations. Not all features of the RHpE could be assessed for some test designs and test locations. For example, for some tests it was not possible to assess straightness in canter on either one rein or both reins, because the assessor was positioned in one standardised location. Strong wind influenced tail carriage, so under some weather conditions the straightness of the tail could not be assessed. Long grass on occasions prohibited accurate determination of the presence or absence of a toe drag. A behaviour was only determined to be present if this was an unequivocal observation. The observer could not be blinded to horse or rider identity, with the potential for bias, however the horse’s subsequent performance could not be predicted, and all statistical analyses were performed completely independently. The duration of the tests was approximately 5 min, the lower end of the spectrum for accurate application of the RHpE [82]. The BE 90 and 100 tests did not incorporate 10 m diameter circles in trot, which are more biomechanically demanding than 20 m diameter circles, and effective in highlighting gait abnormalities and influencing behaviour [82]. Several judges commented that ‘they found it difficult to mark down professional riders’. There are a variety of factors which may adversely influence dressage scores in addition to lameness, including rider skill [17,19], tack fit for horse and rider [6] and how the horse has been trained [83]. Jumping performance may also be influenced by rider skill, confidence and fitness, the athletic capability of the horse, the difficulty of the course, the weather and the terrain and ground conditions [18]. Despite these limitations, consistent results were acquired, with a large data set, across a wide range of venues and competitors.

## 5. Conclusions

There were significant associations between RHpE scores and performance for horses competing at BE 90, 100 and Novice one-day events. Horses placed in the top three had significantly lower median RHpE scores compared with horses which completed but were not placed in the top three. This indicates that although the quality of performance in one-day events is affected by many factors, musculoskeletal pain is likely to be influential in some horses. Although the median RHpE score was low, supporting the social licence to compete, 9% of competition starters had a RHpE score of ≥8/24, indicating the presence of musculoskeletal pain. Horses with a RHpE score of ≥8/24 performed less well than those with a RHpE score <8. Clinical investigation of horses with pain-related gait abnormalities and instigation of appropriate treatment and management may enhance both welfare and performance. Further education for riders, coaches/trainers and dressage judges is required to facilitate the recognition of signs reflecting pain-related gait abnormalities.

## Figures and Tables

**Table 1:** The frequency of occurrence, expressed as a percentage of competition starts, of forelimb lameness, hindlimb lameness, abnormalities of canter, bilaterally short forelimb steps, a short stepping hindlimb gait which lacked hindlimb impulsion and engagement, stumbling once or stumbling more than once at British Eventing (BE) 90, 100 and Novice competitions. Significant differences among competition levels are highlighted in grey.

Gait Abnormality	Overall (%) *n* = 1010	BE 90 (%) *n* = 204	BE 100 (%) *n* = 450	Novice (%) *n* = 356	Chi-square/Fisher’s Exact *p*-Value * (Cramer’s V)
Forelimb lameness	9.1	16.7	9.1	4.5	<0.001 (0.15)
Hindlimb lameness	8.1	15.7	6.4	5.9	<0.001 (0.14)
Abnormal canter	61.0	75.0	66.2	46.4	<0.001 (0.23)
Short stepping forelimb gait	5.1	4.4	3.8	7.3	0.069
Lacked hindlimb impulsion and engagement	38.1	48.0	40.0	30.1	<0.001 (0.14)
Stumbled once	6.6	10.3	5.1	6.5	0.284
Stumbled more than once	1.7	2.6	2.0	0.8	0.047

* p < 0.007 was used as a cut-off for a significant result after adjusting for multiple comparisons using the Bonferroni correction.

**Table 2:** The proportion of breeds as a percentage of competition starts at 34 British Eventing (BE) 90, 100 and Novice competitions.

Breed	BE 90 (%), *n* = 204	BE 100 (%), *n* = 450	Novice (%), *n* = 356
Warmblood	11.3	20.2	28.9
Warmblood cross	16.6	20.2	18.8
Thoroughbred/Thoroughbred cross	1.0	1.1	1.7
Other crossbred/unknown	13.7	13.1	9.8
Irish Sports Horse	37.8	40.7	39.3
Pony	19.6	4.7	1.4

**Table 3:** Age (median, interquartile range [IQR] and range) and sex distribution, expressed as a percentage, of 841 horses competing in 1010 starts at British Eventing (BE) 90 (*n* = 204), 100 (*n* = 450) and Novice (*n* = 356) levels.

Level	Median Age	IQR	Range	Geldings	Stallions	Mares
BE 90	10	8, 13	5, 19	69.1	0	30.9
BE 100	8	6, 11	5, 22	64.4	0.4	35.1
Novice	8	7, 10	5, 19	70.2	1.1	28.7

**Table 4:** The frequency of occurrence, expressed as a percentage of competition starts, of the 24 behaviours of the Ridden Horse Pain Ethogram at British Eventing (BE) 90, 100 and Novice competitions. Behaviours observed in more than 30% of competition starts are highlighted in bold. Significant differences among competition levels are highlighted in grey.

Behaviour	Overall (%) *n* = 1010	BE 90 (%) *n* = 204	BE 100 (%) *n* = 450	Novice (%) *n* = 356	Chi-Square/Fisher’s Exact *p*-Value * (Cramer’s V)
Head up and down repeatedly	17.2	**30.4**	14.9	12.6	<0.001 (0.18)
Repeated head tilt	**39.8**	**41.7**	**37.3**	**41.9**	0.356
Front of head ≥30° in front of vertical for ≥10 s	5.2	15.7	3.8	0.8	<0.001 (0.25)
Front of head ≥10° behind vertical for ≥10 s	**59.3**	**56.4**	**60.9**	**59.0**	0.546
Repeated side to side movement of head	9.6	16.2	8.4	7.3	0.001 (0.11)
Ears behind vertical ≥5 s	**35.8**	**56.9**	**34.7**	25.3	<0.001 (0.24)
Eyes closed 2–5 s; rapid blinking	1.3	2.5	0.9	1.1	0.270
Sclera exposed repeatedly	6.1	8.3	4.2	7.3	0.067
Intense stare ≥ 5 s	**46.5**	**64.7**	**45.3**	**37.6**	<0.001 (0.20)
Mouth opening with separation of the teeth ≥10 s	28.4	**35.8**	20.9	**33.7**	<0.001 (0.15)
Tongue out repeatedly	7.8	10.3	6.9	7.6	0.317
Bit pulled through to one side, repeatedly	15.8	26.0	15.1	11.0	<0.001 (0.15)
Tail clamped to midline or repeatedly crooked	12.4	12.3	10.0	15.5	0.066
Repeated tail swishing	21.0	17.7	21.8	21.9	0.422
Rushed gait or irregular speed	6.6	7.4	7.6	5.1	0.330
Slow gait	0	0	0	0	-
Repeatedly crooked, on 3 tracks	28.4	**39.2**	18.7	**34.6**	<0.001 (0.20)
Repeated incorrect strike off in canter or repeatedly disunited	3.7	4.4	2.2	5.1	0.085
Spontaneous change of gait	10.3	6.9	5.1	18.8	<0.001 (0.21)
Repeated bilateral hindlimb toe drag and/or stumbling > once	**37.4**	**50.5**	**45.1**	20.2	<0.001 (0.27)
Not following rider’s cues; spooking	2.9	2.9	2.7	3.1	0.936
Reluctant to go forwards (requiring verbal encouragement or repeated kicking), or stopping spontaneously	4.0	6.9	3.1	3.4	0.058
Rearing	0.6	0	0.4	1.1	0.310
Bucking	2.0	1.0	2.7	1.7	0.370

* *p* < 0.002 was used as a cut-off for a significant result after adjusting for multiple comparisons using the Bonferroni correction.

**Table 5:** The proportions of completions, eliminations in any phase, retirements (in showjumping or cross-country) and withdrawals (before show jumping or cross-country), expressed as percentages of competition starts (*n* = 1010) for horses competing at British Eventing 90, 100 or Novice levels.

	BE 90 (%) *n* = 204	BE 100 (%) *n* = 450	Novice (%) *n* = 356
Completed	89.2	83.3	82.6
Eliminated	6.9	6.7	7.0
Retired	1.5	5.8	3.9
Withdrawn	2.5	4.2	6.5

**Table 6:** Eliminations or retirements during showjumping or cross-country related to the number of competition starts, accounting for withdrawals or previous elimination, for British Eventing (BE) 90, 100 and Novice competitions.

Phase	Overall		BE 90		BE 100		Novice	
	Number	%	Number	%	Number	%	Number	%
Showjumping	17/997	1.7	1/204	0.5	9/444	2.0	7/349	2.0
Cross-country	94/945	9.8	16/198	8.1	47/422	11.1	31/325	9.5

## Data Availability

Anonymised data are available from the authors on reasonable request.

## Supplementary Materials

The following supporting information can be downloaded at: https://www.mdpi.com/article/10.3390/ani12050590/s1, Table S1: Summary of the Ridden Horse Pain Ethogram (adapted from Dyson et al. 2018) [1].
