# Supplementary material for: Application of the Ridden Horse Pain Ethogram to Horses Competing in British Eventing 90, 100 and Novice One-Day Events and Comparison with Performance
Source: Animals (Basel). 2022 Feb 25;12(5):590. doi: 10.3390/ani12050590 (PMC8909886; doi:10.3390/ani12050590)
Supplement: Supplementary file 1 [file animals-12-00590-s001.zip › animals-1606594-supplementary.pdf]

Supplementary Materials

Table S1. Summary of the Ridden Horse Pain Ethogram (adapted from Dyson et al. 2018) [1]. Definitions of the 24 behaviours.

1. Repeated changes of head position (up/down), not in rhythm with the trot
2. Head tilted or tilting repeatedly
3. Head in front of vertical (≥30^{0}) for ≥10s
4. Head behind vertical (≥10^{0}) for ≥10s
5. Head position changes regularly, tossed or twisted from side to side, corrected constantly
6. Ears rotated back behind vertical (both or one only) for ≥5s; repeatedly lay flat
7. Eye lids closed or half closed for 2-5 s; frequent blinking
8. Sclera exposed repeatedly
9. Intense stare (glazed expression, 'zoned out') for ≥5s
10. Mouth opening ± shutting repeatedly with separation of teeth, for ≥10 s
11. Tongue exposed, protruding or hanging out, and/or moving in and out repeatedly
12. Bit pulled through the mouth on one side (left or right), repeatedly
13. Tail clamped tightly to middle or held to one side
14. Tail swishing large movements: repeatedly up and down/side to side/ circular; repeatedly during transitions, but not in synchrony with spur cues
15. A rushed gait (frequency of trot steps > 40/15s); irregular rhythm in trot or canter; repeated changes of speed in trot or canter
16. Gait too slow (frequency of trot steps <35/15s); passage-like trot
17. Hindlimbs do not follow tracks of forelimbs but repeatedly deviated to left or right; on 3 tracks in trot or canter
18. Canter repeated leg changes change of leg in front and / or behind; repeated strike off wrong leg; disunited
19. Spontaneous changes of gait (e.g., breaks from canter to trot or trot to canter)
20. Stumbles or trips more than once; repeated bilateral hindlimb toe drag
21. Sudden change of direction, against rider's direction; spooking
22. Reluctance to move forwards (has to be kicked ± verbal encouragement), stops spontaneously
23. Rearing (both forelimbs off the ground)
24. Bucking or kicking backwards (one or both hindlimbs)

Reprinted from J. Vet. Behav. Clin. Appl. Res., *23*, Dyson, S., Berger, J., Ellis, A., Mullard, J., Development of an ethogram for a pain scoring system in ridden horses and its application to determine the presence of musculoskeletal pain, Page 53, Copyright (2018), with permission from Elsevier.
